# Virtual-'Light-Sheet' Single-Molecule Localisation Microscopy Enables Quantitative Optical Sectioning for Super-Resolution Imaging

**DOI:** 10.1371/journal.pone.0125438

**Published:** 2015-04-17

**Authors:** Matthieu Palayret, Helen Armes, Srinjan Basu, Adam T. Watson, Alex Herbert, David Lando, Thomas J. Etheridge, Ulrike Endesfelder, Mike Heilemann, Ernest Laue, Antony M. Carr, David Klenerman, Steven F. Lee

**Affiliations:** 1 Department of Chemistry, University of Cambridge, Lensfield Road, Cambridge, CB2 1EW, United Kingdom; 2 Genome Damage and Stability Centre, University of Sussex, Falmer, Sussex, BN1 9RQ, United Kingdom; 3 Department of Biochemistry, University of Cambridge, 80 Tennis Court Road, Cambridge, CB2 1GA, United Kingdom; 4 Institute of Physical and Theoretical Chemistry, Goethe University Frankfurt, Max-von-Laue-Str. 7, 60438, Frankfurt, Germany; Tufts University, UNITED STATES

## Abstract

Single-molecule super-resolution microscopy allows imaging of fluorescently-tagged proteins in live cells with a precision well below that of the diffraction limit. Here, we demonstrate 3D sectioning with single-molecule super-resolution microscopy by making use of the fitting information that is usually discarded to reject fluorophores that emit from above or below a virtual-'light-sheet', a thin volume centred on the focal plane of the microscope. We describe an easy-to-use routine (implemented as an open-source ImageJ plug-in) to quickly analyse a calibration sample to define and use such a virtual light-sheet. In addition, the plug-in is easily usable on almost any existing 2D super-resolution instrumentation. This optical sectioning of super-resolution images is achieved by applying well-characterised width and amplitude thresholds to diffraction-limited spots that can be used to tune the thickness of the virtual light-sheet. This allows qualitative and quantitative imaging improvements: by rejecting out-of-focus fluorophores, the super-resolution image gains contrast and local features may be revealed; by retaining only fluorophores close to the focal plane, virtual-'light-sheet' single-molecule localisation microscopy improves the probability that all emitting fluorophores will be detected, fitted and quantitatively evaluated.

## Introduction

Research in fluorescence microscopy is driven by the continuous effort to increase image quality, and to maximise the information output. A robust and successful approach has been to confine either the excitation or detection geometries to reduce the background light emitted by out-of-focus fluorophores and consequently increase the contrast in the final image. These strategies have resulted in techniques such as: two photon [1], total internal reflection (TIRF) [2], highly inclined and laminated optical sheet (HiLo) [3], and single plane illumination microscopy (SPIM, more generally referred as light-sheet fluorescence microscopy) [4] for spatially limiting the illumination field to the focal plane, and confocal and spinning disk confocal microscopy for spatially filtering the in-focus emitted light.

Independently of contrast optimisation, super-resolution fluorescence microscopy has been developed to overcome the Abbe diffraction-limit of light [5]. Many single-molecule localisation microscopy (SMLM) methods have been described, such as (fluorescence) photo-activation localisation microscopy ((f)PALM) [6,7] or (direct) stochastic optical reconstruction microscopy ((d)STORM) [8,9], which separate densely-packed single emitters in time by stochastically switching their fluorescent states. Each emitter is then observed as a diffracted spot, or point-spread function (PSF), on the detector of the microscope. The PSF is then fitted, typically with a 2D-Gaussian function and the image reconstructed point-by-point, with another 2D-Gaussian, whose width is proportional to the positional precision of the original localisation. Although 3D SMLM techniques have been developed [10–13], most of which are based on the axial variation of the PSF [14], they substantially increase the complexity of imaging and so have not yet been widely adopted by researchers that are using SMLM imaging to address fundamental biological problems. Similarly, SMLM has been successfully combined to light-sheet illumination [15], although some geometrical challenges still prevent its general adoption [16].

Nevertheless, some 3D information is inherently retained in 2D SMLM data. Here we present a simple method using the experimentally-determined axial variation of the PSF of any microscope to selectively identify fluorophores that are located within a defined thin volume, or virtual-‘light-sheet’ (vls), centred on the focal plane of the microscope. Conceptually, this method can be compared to light-sheet illumination: both allow an increase in contrast in the final image by eliminating fluorophores outside of a thin focal plane. But instead of spatially limiting the illumination field to a thin focal plane, we computationally apply a virtual pinhole (or width threshold) to each fitted emitter. This process is analogous to placing a physical pinhole in the image plane to reject out-of-focus emitted light in confocal microscopy, which is not physically possible in wide-field SMLM techniques. This method, based on post-PSF-fitting thresholding, does not require any additional optics and is applied independently of the PSF-fitting algorithm used. It can be easily applied to existing complex 2D data and instrumentation. It extracts quantitative information from the fitted parameters of each individual emitter that are usually discarded or arbitrarily used. Tuning the thickness of the vls allows for increased contrast in the final super-resolved image in qualitative experiments, prevents an undercounting bias in quantitative experiments, and increases certainty in 3D bio-imaging. It is of particular interest in the case of imaging 3D volumes for which increased contrast is important, though difficult to obtain by structured illumination geometries (*e*.*g*. in the nucleus of living cells).

## Results

### Characteristics of the PSF

**Fig 1:**
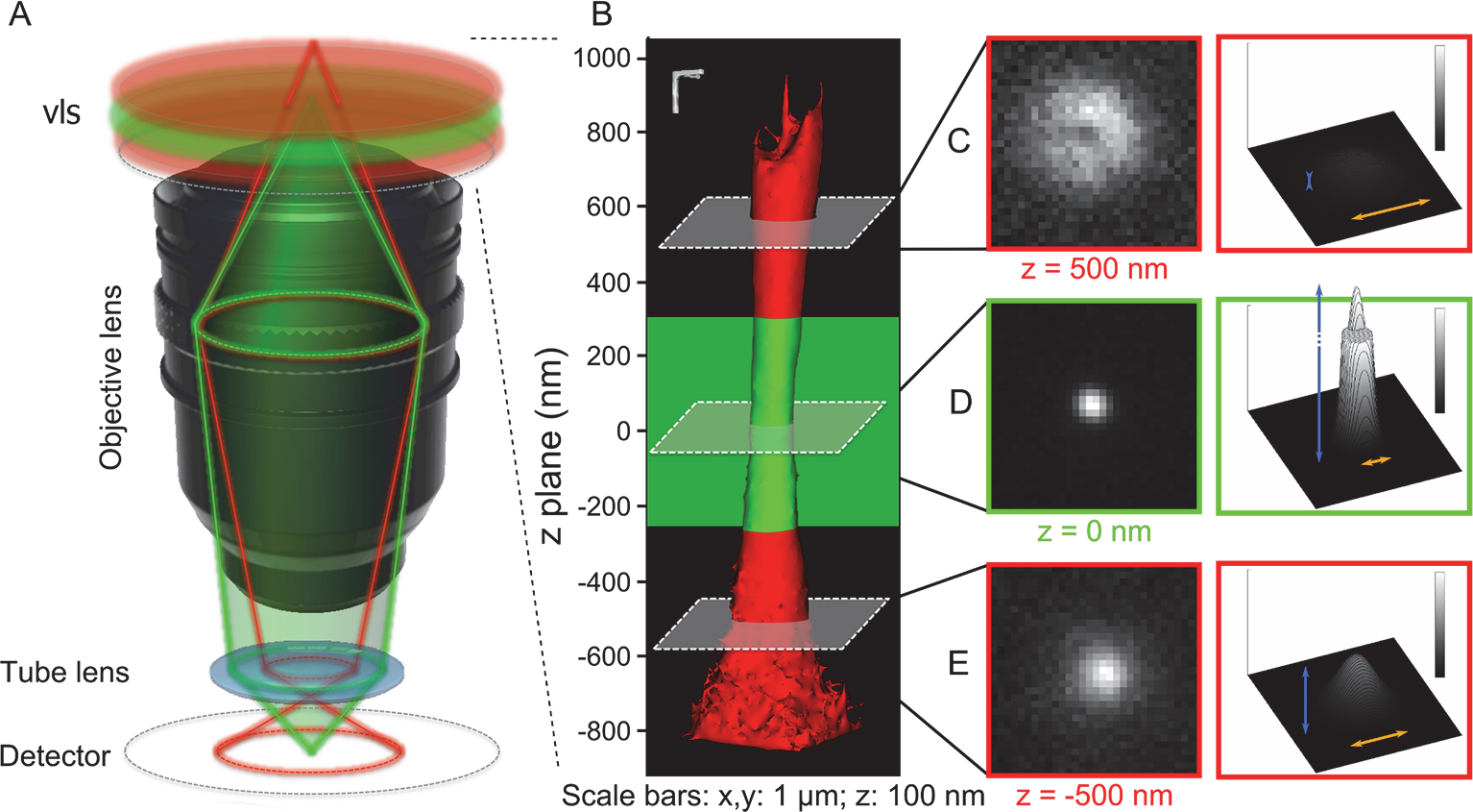
Variation of the PSF in three dimensions. A vls (green plane, **A**) is defined as a volume above and below the focal plane of the microscope from which an emitter is imaged as a diffraction-limited spot on the detector (green optical path, **A**). A fluorophore emitting from outside the vls (red volume above and below the vls, **A**), is blurred on the image plane of the detector (red optical path, **A**). The z-stacks of 28 sub-diffraction beads were superposed to image the axial variation of the PSF of the instrument. The contrast-adjusted rendered volume **(B)** of the z-stack shows the axial variation of the width of the PSF. Three examples of (xy) planes of the z-stack in, above and below the vls are shown in **(C-E)**. For each plane, a contrast-adjusted image (left column) and an intensity surface plot (right column) of the plane underlines the axial variation of the width (orange arrows) and the amplitude (blue arrows) of the PSF.

SMLM methods overcome the diffraction limit of light by stochastically separating the emission of single fluorophores in time and, one by one, fitting their PSF in order to localise them with a precision proportional to $1/\sqrt{N}$, where N is the number of photons collected [17]. Such methods typically achieve localisation precisions of ~10 nm or lower in fixed cells, an order of magnitude better than conventional diffraction-limited systems. The PSF of a point-emitter is the impulse response of the optical system (composed of the objective and tube lenses), and is manifested as a diffraction-blurred punctum in the image plane, which is typically detected by an EMCCD [18] / sCMOS [19] camera (Fig 1A, green path). In standard wide-field microscope setups, the PSF of a point emitter is well approximated with a 2D-Gaussian [20] in the conjugate plane of the emitter. An emitter originating close to the focal plane of the imaging system will consequently be observed on the camera as a diffraction-limited PSF, well approximated with a 2D-Gaussian. However, an out-of-focus point emitter—that is, a single fluorophore whose position lies outside the focal plane—will have its conjugate plane located in front of (or behind) the plane of the camera, and as a result its PSF will be observed larger on the camera (Fig 1A, red path).

In fluorescence microscopy, the image can be understood as the convolution of the signal emitted by all the individual point emitters with the PSF of the microscope. The image includes molecules outside of the focal plane, which contribute to a ‘diffuse blur’ that increases the fluorescence background and hence decreases contrast. By only collecting light through a pinhole placed at the image plane, confocal microscopy rejects this out-of-focus background *physically*. However, in wide-field non-scanning techniques such as SMLM, a pinhole should not be used. But in 2D SMLM methods, as PSFs are imaged and fitted individually one at a time, their widths are known and each PSF can be *computationally* selected depending on their width, as a pinhole would. In doing so, a vls is generated by either rejecting or including individual localisations in the final image reconstruction for improved optical sectioning. In the final image, only fluorophores within the vls will be visible, as if the sample had been illuminated by a thin sheet of light.

### Defining the bounds of the virtual-‘light-sheet’

In order to implement vlsSMLM, one needs to first characterise the optical properties of the PSF of each microscope. To do this, a 10 nm z-stack of 40 nm fluorescent beads was imaged to observe the axial variation of the 2D-PSF. We confirmed that sub-diffracted beads, under low illumination, mimic idealised single fluorophores (S1 Fig). Compared to single fluorophores, beads can be imaged fixed on the coverslip for multiple frames at different axial positions without photo-bleaching. The PSFs of 28 beads were averaged (Fig 1B) to obtain a finer model of the PSF. The mean position of the focal plane was defined as the plane for which the integrated intensity of a box drawn around the 2D-PSF was the highest. Around this plane, the vls (green slice in Fig 1B) is created as an arbitrary volume above and below this point. Due to the axial resolution of most high-numerical aperture super-resolution microscopes, the height of the vls is typically defined as ~600 nm (full width at half maximum); in our experiment, the variation of the integrated intensity of the 2D-PSF along the optical axis could be fitted with a Gaussian function with a full width at half maximum of 666 nm (corresponding to a standard deviation σ = 283 nm).

As expected, a clear axial variation in both amplitude and width was detected (Fig 1C–1E). From all studied parameters, amplitude and width were those that varied the most in magnitude along the z-axis and the least in the corresponding (xy) plane (S2 Fig and S1 Discussion). Therefore, in an SMLM experiment, we can reject out-of-focus PSFs by simply applying thresholds to the fitted width and amplitude of each localisation, consequently increasing the contrast in the super-resolved picture. We decided to study the effect of such thresholds in relation to the vls in a controlled and quantified manner.

### Building the virtual-‘light-sheet’

To build the vls, the images of fluorescent beads in the previous z-stack were used individually as idealised non-bleaching single fluorophores, imaged in and out of the defined vls volume. The laser power was adapted so that the beads emit a similar photon fluence to single fluorophores (between 500 and 1,500 photons detected per frame). In this system, the axial positions of the fluorescent beads are precisely known in the different frames of the z-scan and this experiment allows determination of the optimal thresholds to apply to actual SMLM data when the relative positions of the emitters to the vls are to be determined.

**Fig 2:**
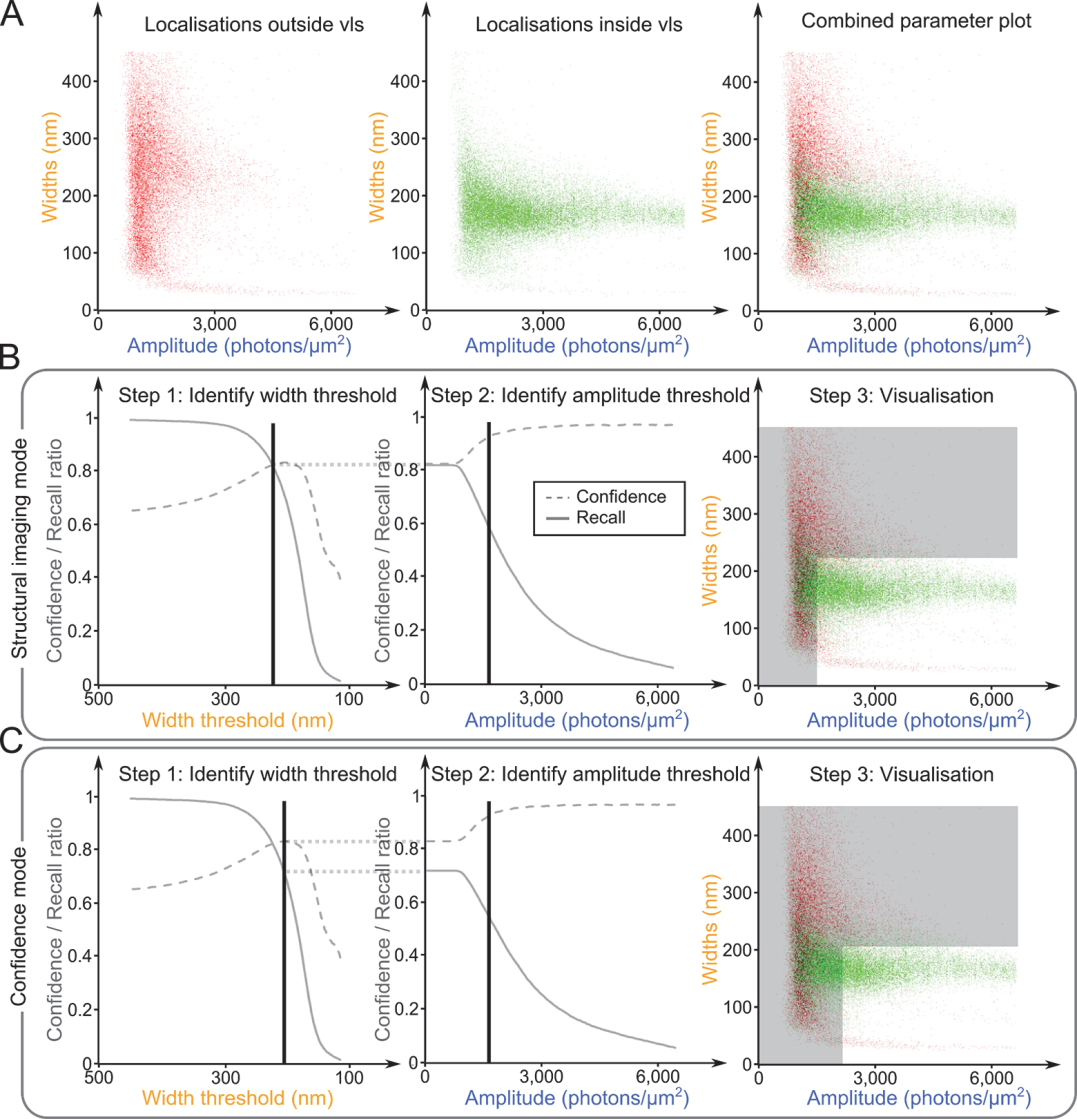
Building the virtual-‘light-sheet’. A calibration z-stack of images of immobile and separated, sub-diffraction fluorophores is imaged and its 2D-PSFs fitted. For each fitted PSF, the width is plotted against the amplitude in a parameter plot **(A)**. PSFs detected in planes from within the vls are plotted in green; PSFs coming from outside of the vls are plotted in red. Two imaging modes are described, aimed at two different SMLM analyses: the structural mode **(B)** aims to increase the contrast of the image without rejecting a majority of localisations; the confidence mode **(C)**, to reject more localisations and only accept localisations from within the vls with a higher certainty. For each mode, three steps are described: first the confidence (dotted grey line) and the recall (solid grey line) ratios are calculated for different width thresholds (left column). A width threshold (black vertical line) is identified to optimise both ratios in accordance with the aim of the experiment of interest. This step is repeated with the new thresholded list of localisations to identify an amplitude threshold (middle column). Finally, both chosen thresholds are visualised on the parameter plot (PSFs in the grey areas are rejected).

The PSF from each bead in each frame was extracted and fitted using Peak Fit [21], an algorithm which fits a 2D-Gaussian to each PSF and returns five parameters: the *(x*, *y)* position of its centre, its width, its amplitude and its offset. As observed when characterising the axial change of the 2D-PSF of the microscope (Fig 1C–1E), the two parameters that changed the most between individual PSFs emitting from inside or outside of the vls were the width and amplitude (S2 Fig and S1 Discussion). For each localisation detected, these two parameters were plotted against each other in false colour representing whether the single PSF detected originated from within (green) or outside (red) the vls (Fig 2A). We henceforth refer to this as the parameter plot.

We next investigated each of these two PSF-fitting parameters in a two-step process: we first optimised and fixed the width threshold before identifying a second amplitude threshold to apply (Fig 2). Indeed, the amplitude of the PSF of a fluorophore does not depend only on whether the fluorophore is in the vls but also on many different parameters such as its (xy) position in the inhomogeneous illumination field, which fluorescent states it occupies, or the orientation of its dipole [22] (S1 Discussion). The width of a PSF, on the contrary, depends only on its axial position, and its diffusion during the exposure time of the frame [23]. Therefore, the width parameter is less convolved with other phenomena than the axial position of the emitter and consequently allows better discrimination between emitters that are inside and outside of the vls.

First, the effect of applying a width threshold to the fitted PSFs was studied. A threshold eliminating all PSFs with widths larger than a value varying from 500 to 100 nm was applied. For each threshold, the confidence and the recall ratios were calculated as follows:

$$confidence=\frac{TP}{TP+FP},\;\;\text{and}\;\;call=\frac{TP}{TP+FN},$$

where *TP* represents the true positives (green points kept after thresholding), *FP* the false positives (red points kept after thresholding), and *FN* the false negatives (green points that have been thresholded out). The confidence ratio is the probability that a PSF that has been kept after thresholding actually comes from the in-focus volume. The recall ratio measures the fraction of emitters from the in-focus volume that are kept after thresholding.

As the threshold increases, more and more PSFs are eliminated and the recall metric drops (Fig 2B and 2C, left panels, solid lines). However, most of the eliminated PSFs come from the out-of-focus volume, thus the confidence increases until a maximum value is obtained (Fig 2B and 2C, left panels, dashed lines). In order to choose a width threshold, a compromise needs to be made between high confidence (to increase the contrast of the final rebuilt picture) and a high enough recall to correctly sample the structure of interest.

We then calculated the confidence and recall ratios for different amplitude thresholds, applied after the width threshold previously chosen (Fig 2B and 2C, middle panels). Again, we adjusted the amplitude threshold to obtain a high confidence ratio together with a high enough recall rate. Since the beads were imaged under conditions for which their intensities were similar to the intensities of single fluorophores in SMLM experiments, similar amplitude thresholds can be used for the analysis of SMLM experiments (S1 Fig).

Finally, the result of applied thresholds can be observed on the parameter plots (Fig 2B and 2C, right panels). The aim of the thresholding is to select the concentration of green points with widths of around 175 nm (for our 505-nm-emitting beads), while eliminating most of the red points.

### Application of the vlsSMLM to super-resolution imaging of cellular structures

We propose two different vlsSMLM imaging modes for two different potential applications in vlsSMLM. The first—‘*structural imaging mode’* (Fig 2B) allows controlled enhancement of contrast, while retaining as many of the fitted localisations as possible, in order to maintain the sampling resolution in structures of interest. The width threshold for this mode was defined as the intersection of the confidence and recall curves (223 nm). We then applied an amplitude threshold that gave a confidence ratio of 90% for the structural imaging mode (1,502 photons/μm^2^). The second imaging mode—*‘confidence mode’* (Fig 2C), favours the confidence ratio over the recall, to be sure that the retained localisations are inside the vls volume. The width threshold was thus defined as the width for which the confidence ratio is maximal (206 nm). The amplitude threshold was then fixed to obtain a confidence ratio of 95% (2,164 photons/μm^2^).

To illustrate the two imaging modes, we imaged two different biological structures and applied vlsPALM both to increase contrast and to look at the distribution of 3D clusters. These examples are chosen to exemplify the types of problem addressable by the different imaging modes. In both examples, the middle planes of the cellular structures of interest (the yeast vacuoles or the nucleus of a mammalian stem cell) were positioned more than 100 nm above the coverslip, *i*.*e*. above the possible TIRF field. To image such structures, if a microscope with light-sheet capability is unavailable, a HiLo [3] illumination has to be used. Such broad illumination excites a large proportion of out-of-focus fluorophores which contribute to an increased fluorescent background. We applied vlsPALM thresholds to reject these out-of-focus fluorophores.

### Structural imaging mode

**Fig 3:**
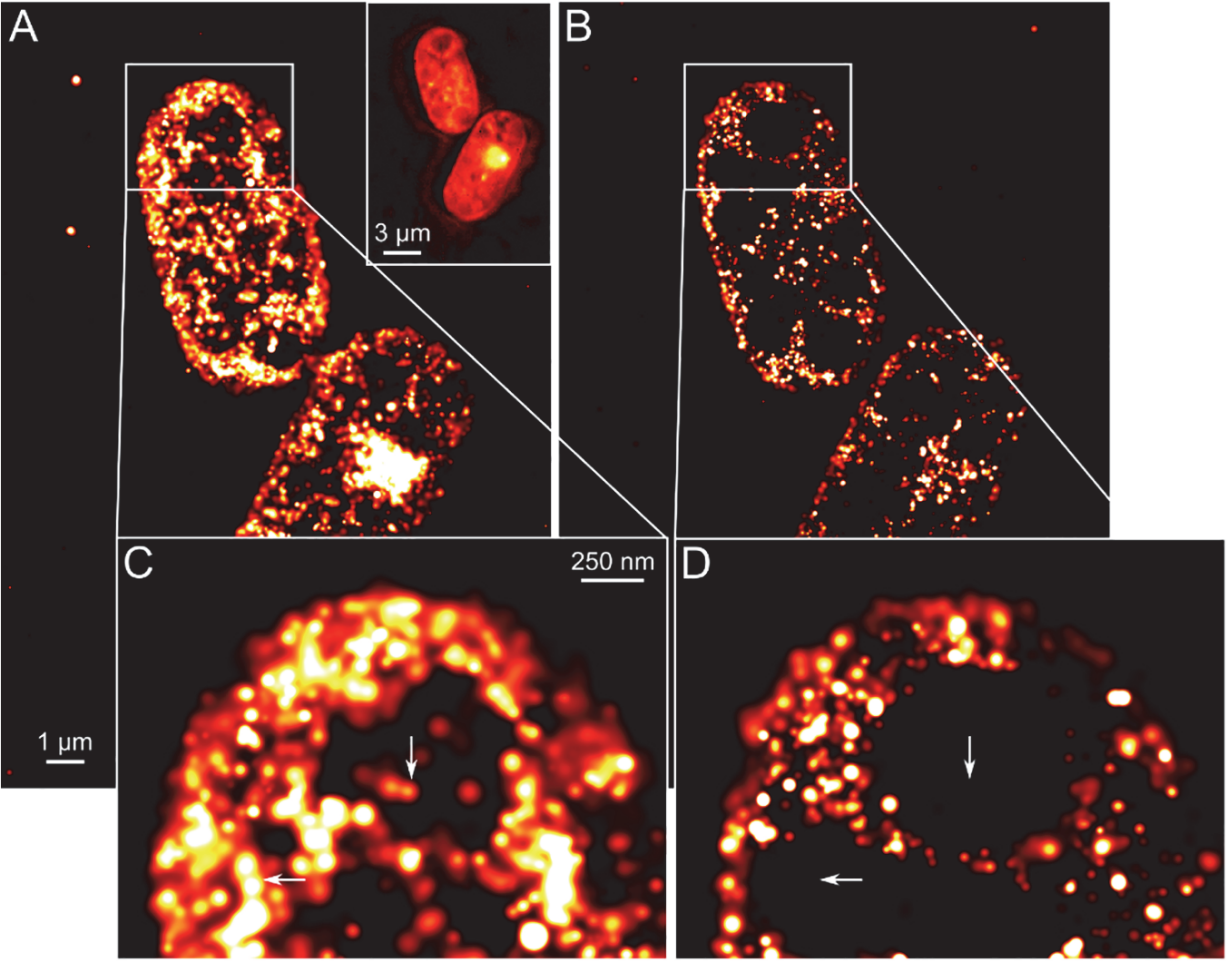
Structural imaging mode. Fixed *S*. *pombe* expressing cytoplasmic Cdc22-mEos proteins were imaged during a PALM experiment. 5,000 frames were analysed with Peak Fit and the resulting list of localisations was used to produce a super-resolved picture directly after fitting **(A)**, or after applying the vlsPALM thresholds defined in Fig 2B **(B)**. The corresponding diffraction-limited image of the two cells is shown as an inset in **(A)**. Close-ups of the white rectangles in **(A-B)** are shown in **(C-D)**. The contrast of the large intracellular vesicles of the yeast is increased after vlsPALM filtering (white arrows in **(C-D)**).

We imaged fixed fission yeast cells expressing the cytosolic protein Cdc22 fused to mEos2 at its C-terminus, to demonstrate the increase in contrast that the structural imaging mode allows and reveal a cellular organelle. The fission yeast *Schizosaccharomyces pombe* (*S*. *pombe*) is a powerful and highly tractable eukaryotic model organism, often used to study the cellular responses to DNA damage and the process of DNA replication. In response to nitrogen starvation or to osmotic stress, large vacuoles appear in *S*. *pombe* cells in order to restore the concentration of the cytosol [24]. We used Cdc22, a protein which is highly expressed and freely diffusive in the cytoplasm of the yeast, to image, in contrast, this organelle. Applying the vlsPALM thresholds in structural imaging mode, an increase in contrast is observed in the super-resolved image (Fig 3B and 3D): some vacuoles only appear after thresholding as out-of-focus PSFs from below or above the vacuoles are detected and plotted in the non-thresholded picture (Fig 3A and 3C).

### Confidence mode

**Fig 4:**
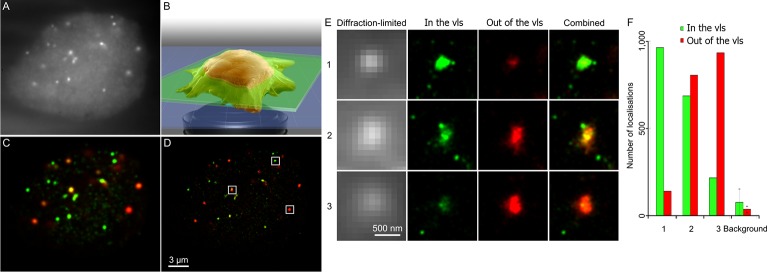
Confidence mode. Embryonic stem cells expressing Cenp-A-mEos proteins were fixed and imaged. The corresponding movie (summed in **A**) was analysed with Peak Fit and the resulting list of localisations was separated between in vls (green) and out of vls (red) localisations using the vlsPALM thresholds defined in Fig 2C. vlsPALM allows the identification of the in-focus localisations **(B)**. All localisations were plotted either as fitted **(C)** or in a super-resolved picture **(D)**, but coloured according to the vlsPALM filtering. Three categories of Cenp-A clusters were observed: some were almost entirely within the vls (**D-F**, 1); others were spanning one extremity of the vls, partly in the vls (**D-F**, 2); the last ones were entirely out of the vls (**D-F**, 3). **(E)** shows the diffraction-limited and super-resolved close-ups of the Cenp-A clusters defined in **(D)**. **(F)** displays the number of localisations in (green) and out of (red) the vls for each cluster. Such classification allows selecting in-focus clusters for further quantification and preventing under-counting due to undetectable out-of-focus emitters.

We used fixed mouse embryonic stem cells stably expressing mEos3.2-tagged centromere protein A (Cenp-A) that form distinctive clusters to show how quantification of super-resolved clusters benefits from the confidence mode of the vlsPALM analysis. Cenp-A is a histone H3-like protein that is present in nucleosomes at the centromeres in eukaryotic cells. Cenp-A forms foci at a number of defined points in a nucleus and determining the structure of such foci or their stoichiometry is of major interest in the yeast genetics field [25]. However, out-of-focus fluorophores have larger and dimmer PSFs (Fig 1), making them more difficult to detect and fit over the background. Thus, only clusters which are within the focal plane will have all their fluorophores correctly detected, while those outside the focal plane will have part of their fluorophores undetected. Using vlsPALM (Fig 4A–4D) allowed us to select only in-focus clusters (Fig 4E) in order to analyse them for further quantification (Fig 4F), preventing any initial under-counting.

## Discussion

In this study, we present a simple, cost-effective, powerful method for selecting in-focus fluorophores to increase the contrast in super-resolved images that can be used in conjunction with any 2D super-resolution microscope and any super-resolution PSF-fitting algorithm. This method however is incompatible with basic centroid-finder algorithms that give no precise information about width and amplitude of the localisations. By virtue of the fact that each PSF is collected, one can tune a thin, in-focus slice from a super-resolved image, and in doing so create a virtual-‘light-sheet’. Most fitting codes already apply arbitrary thresholds, but these are generally used as a fitting check, not as a quantitative method to get more information from the detected single molecules. In our experiments using fluorescent proteins (mEos2 and mEos3.2), which emit at approximately 580 nm, we determined that a threshold of 220 nm for width (standard deviation of the Gaussian fit) and 1,500 photons/μm^2^ for amplitude increased the contrast of our super-resolved images, allowing the observation of cellular organelles and the detection of in-focus protein clusters.

This method only requires a simple z-scan calibration of fluorescent beads, normally achieved via a controlled z piezo-driven stage (see Material and Methods, Beads and PALM imaging). The calibration is used to build up a ‘parameter-plot’ (Fig 2A and S1 Discussion) that is specific for each individual imaging system or microscope, and then to define thresholds with known confidence and recall rates that can then be applied to the SMLM data. We show that the calibration does not require extensive sampling: as few as 31 localisations per axial step (next to the focal plane) and steps of up to 40 nm did not quantitatively affect our results (S3 Fig). Also virtually any fixed and separated sub-diffraction fluorescent point source (*e*.*g*. beads, gold particles or quantum dots) can be used for the calibration. The calibration proved to be robust to very different protocols on a number of different instruments tested so far (S4 Fig). However, the actual values of the vlsSMLM thresholds both depend on the wavelength of the fluorophores and on the specific optics used. Thus, a calibration should be measured on the same instrument that is used for the actual SMLM experiment with beads whose spectrum is matched to the wavelength of the fluorophore used in the SMLM experiment.

We implemented this method as a freely available ImageJ plug-in [26], which acts as a post-processing layer that can use any 2D-Gaussian fitting results (S5 Fig) and is thus independent of the fitting algorithm. The actual values of the vlsSMLM thresholds are nevertheless dependent on the fitting algorithm used for fitting both calibration and SMLM data. The calibration step should thus be analysed with the specific fitting routine used to fit the SMLM data. This vlsSMLM plug-in is included in the supplementary information (S1 Software, source-code available at https://github.com/MatthieuPalayret/vlsSMLM). It offers two functions: the first uses a calibration z-scan to calculate (and display) parameter plots with the corresponding confidence and recall rates specific to the instrument used, as shown in Fig 2B and 2C. The second function takes the list of fits from a SMLM experiment and interactively displays a super-resolved image with user-defined thresholds. It also outputs the list of thresholded fits for further analysis or reconstruction (see full documentation for more details). The vlsSMLM plug-in has been designed to be a user-friendly tool and is available both as a stand-alone ImageJ plug-in or directly integrated into the Peak Fit plug-in [21], a quick efficient SMLM fitting algorithm, making the vlsSMLM plug-in able to analyse any SMLM stack without any other pre-processing PSF-fitting step.

As in light-sheet illumination, vlsSMLM imaging allows an increase in contrast in the final super-resolved image, although any illumination geometry can be used (TIRF, HiLo or epifluorescence). Moreover, the width of the focal volume, or vls, can be tuned by changing the thresholds applied. However, although a main advantage of light-sheet illumination is the minimisation of photo-bleaching of the fluorophores and photo-damage of the sample as only the imaged plane is illuminated, vlsSMLM imaging does not reduce photo-bleaching or photo-damage as it is a post-process analysis. It is therefore not suitable for experiments requiring multiple successive axial slicing to observe multiple planes in a sample.

## Conclusions

vlsSMLM is an easy-to-use filtering ImageJ plug-in that only requires a z-scan movie of immobile, sub-diffraction and separated fluorescent particles for calibration. Using any fitting code that provides information about the width and amplitude of each detected fluorophores allows thresholds to be chosen for use in further experiments. In SMLM experiments, as PSFs are obtained one-by-one, information about whether each emitter resides within the vlsSMLM is known. With the vlsSMLM plug-in, one can quantitatively reject fluorophores outside the vls and thereby also qualitatively increase the contrast of super-resolved images. However, as vlsSMLM does not modify the illumination of the sample, it does not limit photo-damage or photo-bleaching as in a physical light-sheet. Nonetheless in cases where axial discrimination is required for localisations close to the focal plane of an objective lens, it provides a simple solution which we hope current users of SMLM will quickly adopt to address important biological questions.

## Material and Methods

### Beads

505 nm emitting 40-nm-diameter beads (Molecular Probes, FluoSpheres NeutrAvidin-Labeled Microspheres, F-8771) were sonicated for 30 s and diluted 1/100 in MilliQ water. The diluted beads were then sonicated for 10 minutes and diluted again 1/1,000 in MilliQ water. A coverslip was argon-plasma cleaned and an imaging hydrophobic gasket (Bio-rad, SLF0201) was applied onto it. It was then coated with 100 μL of fresh sterile poly-l-lysine (Sigma, P4832) for 15 minutes at room temperature, before being washed three times with 100 μL of MilliQ water. 50 μL of the diluted beads were dropped onto the coverslip for 10 minutes at room temperature, washed three times with 100 μL of MilliQ water and imaged with a TIRF setup.

### *S. pombe*

The mEos2 gene sequence was sub-cloned into the pFA6a-kanMX6 C-terminal gene tagging plasmid [27] to create pFA6a-mEos2-kanMX6. The mEos2-kanMX6 sequence was then integrated directly downstream of the *S*. *pombe* cdc22 gene ORF essentially as described [27] to create AW709 (h-, cdc22-mEos2-kanMX6, leu1-32, ura4D18). A PCR against the inserted fragment showed that the diploid cells were heterozygote for cdc22-mEos2.

Cells were grown from frozen stocks on YEA (yeast extract 0.5% w/v, glucose 3% w/v, adenine, leucine and uracil all 225 mg/L and 2% Agar) plates then cultured in Edinburgh Minimal Media (EMM2) (filter sterilised) in a shaking incubator at 30°C. Fresh plates were prepared for new experiments and the cultures set up 12–24 hours before the experiment. Samples for fixing were taken at an OD595 of 0.1–0.2. Samples were spun down at 8,000 g. The cell pellets were fixed in 1% formaldehyde (made from 16% methanol free stock, Fisher Scientific) in MilliQ water at room temperature for 15 minutes. The fixed sample was spun down again and washed three times in MilliQ water before being re-suspended in 10–20 μL of MilliQ water.

All coverslips and slides were cleaned with an ozone generator and a UV light source. 5 μL of the re-suspended sample were added between a 1% agarose (Sigma, A0169) pad (100 μL of 1% agarose in MilliQ water pipetted between two 20 x 20 mm coverslips) and a cleaned slide.

### Embryonic stem cells

mEos3.2 (mEso3.2 plasmid kindly given by Tao Xu [28]) was first cloned onto the N-terminus of Cenp-A (cDNA plasmid from Thermo Scientific, clone MMM1013-64851) using a cloning vector. PCR of mEos3.2 and Cenp-A were carried out with primers described in S1 Table. Then mEos3.2-Cenp-A was cloned into the NcoI/XbaI site of a mammalian expression vector (pEF.myc.ER-E2-Crimson; Addgene plasmid 38770) [29]. Sequencing of the vector was carried out using the pEF forward primer and the BGH reverse primer (S1 Table).

Mouse embryonic *Mbd3* ^*Flox/-*^ [30] stem cells were cultured in standard serum and LIF conditions as previously described [31]. These cells were transfected with the mEos3.2-Cenp-A plasmid using lipofectamine 2000 (Life Technologies), and after 24 hours, a stable cell line was selected and maintained using 500 μg/mL geneticin selection. These cells were then trypsinized and resuspended in PBS, fixed in 1% methanol-free formaldehyde (ThermoScientific, 28908) for 5 minutes and then imaged on 22 x 22 mm coverslips.

### PALM imaging

Collimated 561 nm (Cobolt, Jive 200), 488 nm (Toptica, iBeam Smart 488 100 mW), and 405 nm (Oxxius, LaserBoxx 405) laser beams were aligned and focussed at the back aperture of an Olympus 1.49 NA 60x oil objective mounted on an IX71 Olympus inverted microscope frame. The power of the collimated beams at the back aperture of the microscope was respectively 400, 40, and 0.6 W/cm^2^. The fluorescent signal was filtered with a four-band dichroic (Semrock, Di01-R405/488/561/635) and either a 488 long-pass filter (Semrock, BLP01-488R, for beads), or a combination of a 561 long-pass (Semrock, BLP01-561R) and a band-pass filters (Semrock, FF01-593/40 for yeast imaging; Semrock, FF01-587/35 for stem cell imaging), expanded through a 2.5x achromatic beam expander (Olympus, PE 2.5x 125) and finally projected onto an EMCCD (Photometrics, Evolve 512).

For imaging the beads, a z-stack of 201 steps of 10 nm was taken. For each step, 10 frames were imaged with a 33 ms exposure. For both yeast and stem cell imaging 5,000 frames were taken at a 50 ms exposure. In the super-resolution images, each localisation is plotted as a sub-pixel with a specific intensity convolved with a Gaussian with a specific standard deviation. In Fig 3, the intensity of the rebuilt localisation is equal to the integrated intensity of the fitted PSF, and its width, to the precision (as calculated by Mortensen et al. [32]) of the localisation; in Fig 4, the amplitudes of all rebuilt localisations are equal, and their widths correspond to the average precision of all the localisations.

## Supporting Information

S1 Discussion Choice of parameters for vlsSMLM filtering. (DOCX)

S1 Fig Under adapted low illumination, the PSF of sub-diffraction beads is indistinguishable from the PSF of single fluorophores. Single fluorophores (TMR* in orange, purified mEos3.1 in green) were fixed on poly-lysine-coated coverslips and imaged under 561 nm illumination. Very low irradiance at 405 nm activation light was used in order to image only a few separated fluorophores per frame. A z-scan was performed so that different fluorophores at different lateral positions were imaged at each axial position of the stage. After fitting each detected 2D-PSF, we rebuilt the width *vs*. amplitude parameter plot for the localisations detected in the vls and compared it to the one obtained for 505 nm emitting beads (40 nm in diameter) as in Fig 2 (navy blue). The distributions of localisations of the single fluorophores and of the bead in the width *vs*. amplitude parameter plot are very similar. Imaging beads of diameter ≤ 100 nm under low illumination is consequently an easy way to mimic single fluorophores and calibrate vlsSMLM.
(TIF)

S2 Fig Comparison of the axial variations of different parameters. After fitting all detected PSFs from a z-stack of images of beads (Fig 1), the (xz) projection of the sum of 28 bead stacks over 1.6 μm was plotted, **(A)** showing its axial variation in intensity space. Multiple parameters were considered to define the vls: the amplitude, the integrated intensity, the signal-to-noise ratio, the precision, the width or the ellipticity of the localisations **(B)**. Some parameters, such as the signal-to-noise ratio, the intensity or the ellipticity, do not vary axially and cannot be used to define a precise focal plane. In principle multi-parameter vlsSMLM imaging is possible; however, the amplitude, precision and width were best suited to large variation relative to the focal plane. We chose the width and the amplitude as our vlsSMLM thresholds as their variation at a given plane (shades around each curve: +/- one standard deviation of the mean) were slightly smaller and therefore would lead to a more precise vls.
(TIF)

S3 Fig Optimisation of the calibration z-stack. Varying the number of beads fitted per z-step, by changing the number of frames imaged per z-step (A), had no impact on the computed confidence (dotted line) and recall ratios (solid line). As few as 31 localisations per in-focus step are sufficient for a correct sampling of the calibration. However, the size of the step of the z-stack had, as expected, a strong impact on the calibration efficiency (B): the confidence rate (dotted line) dropped importantly as soon as the sampling involved z-steps of more than 40 nm. The recall rate (solid line) was flatter, but also dropped for higher z-step sizes of 80 nm or more. We therefore recommend sampling the calibration beads at least every 40 nm along the optical axis; this can be achieved in multiple ways including piezo, mechanical or even manual positioning.
(TIF)

S4 Fig vlsSMLM analysis is robust to using both different instrumentations and different fluorescent particles to evaluate the PSF. Independent z-scans of different isolated fluorescent particles were imaged on different instruments to test the robustness of the vlsSMLM calibration. Analyses of four z-stacks (see S1 Material and Methods) were compared to the z-stack described in the Results section (see Material and Methods): one frame of 505 nm, 560 nm, or 660 nm emitting beads, or 655 nm emitting quantum dots was imaged every 20 nm on three different instruments; ten frames of 560 nm emitting beads were also imaged every 40 nm with a water immersion 1.2 numerical-aperture objective. The thickness of the vls is anti-correlated with the numerical-aperture objective lens [33]: for the 1.2 numerical-aperture objective, the thickness of the vls was increased to σ = 2,000 nm instead of 283 nm. The parameter plots (**A**) look relatively similar, although they vary in density; the PSFs from the vls are more dense below the 223 nm width threshold (black horizontal line). Black vertical lines correspond to the 1,502 photons/μm^2^ amplitude threshold. Similarly, the variations of the confidence (**B**) and the recall (**C**) ratios qualitatively follow a similar shape; they however peak (**B**) and drop (**C**) at slightly different threshold values depending on both the wavelength of the fluorescent particles and on the specific instrument. This behaviour is experimentally expected as the width of the PSF is wavelength-dependent [20] and because of the imperfect optics used that differently affect the width of the PSF. A calibration with particles whose spectrum is matched with the spectrum of the fluorophores used in the SMLM experiment is therefore recommended, on the very same microscope. Interestingly, the confidence ratio for the 560-emitting beads experiment is globally lower. This is due to some diffusing beads in solution which are out of the vls but are considered in it during the calibration.
(TIF)

S5 Fig The concept of vlsSMLM post-processing is independent of the fitting code used. Different, widely used, fitting algorithms were used on the same z-stack of images of beads: Peak Fit [21], QuickPALM [34], rapidSTORM [35], ThunderSTORM [36] and M2LE [37]. Their difference is reflected in the parameter plots that were calculated (**A**). However, in-focus PSFs (green parameter plots, **A**) are always found in majority just below the 223 nm width threshold (black horizontal line). The confidence (**B**) and recall (**C**) ratio curves for the different fitting algorithms follow a similar shape. They however peak (**B**) and drop (**C**) at different values, underlining the importance of analysing the calibration z-scan in the very same way as the SMLM data, using the same fitting routine. The 223 nm width and 1,502 photons/μm^2^ amplitude thresholds are shown as black horizontal and vertical lines. Outlier behaviour is noticeable for QuickPALM. This could be explained by a low ability of QuickPALM to precisely fit the widths of the PSFs.
(TIF)

S1 Material and Methods Supplementary Material and Methods. (DOCX)

S1 Software Virtual light-sheet single-molecule localisation microscopy ImageJ plug-in. (ZIP)

S2 Software Section of the SMLM movie analysed in Fig 3 as a dataset to test the vlsSMLM plugin (S1 Software). (ZIP)

S1 Table DNA primers used for Cenp-A-mEos3.2 cloning and sequencing. (DOCX)
