# Supplementary material for: Virtual-'Light-Sheet' Single-Molecule Localisation Microscopy Enables Quantitative Optical Sectioning for Super-Resolution Imaging
Source: PLoS One. 2015 Apr 17;10(4):e0125438. doi: 10.1371/journal.pone.0125438 (PMC4401716; doi:10.1371/journal.pone.0125438)
Supplement: S1 Discussion — (DOCX) [file pone.0125438.s001.docx]

# Virtual-‘Light-Sheet’ Single-Molecule Localisation Microscopy Enables Quantitative Optical Sectioning for Super-Resolution Imaging.

Matthieu Palayret^1^, Helen Armes^1,2^, Srinjan Basu^3^, Adam T Watson^2^, Alex Herbert^2^, David Lando^3^, Thomas J Etheridge^2^, Ulrike Endesfelder^4^, Mike Heilemann^4^, Ernest Laue^3^, Antony M Carr^2^, David Klenerman^1^, Steven F Lee^1*^

^1^ Department of Chemistry, University of Cambridge, Lensfield Road, Cambridge CB2 1EW, UK

^2^ Genome Damage and Stability Centre, University of Sussex, Falmer, Sussex BN1 9RQ, UK

^3^ Department of Biochemistry, University of Cambridge, 80 Tennis Court Road, Cambridge CB2 1GA, UK

^4^ Institute of Physical and Theoretical Chemistry, Goethe University Frankfurt, Max-von-Laue-Str. 7, 60438 Frankfurt, Germany

* [sl591@cam.ac.uk](mailto:sl591@cam.ac.uk)

S1 Discussion: **Choice of parameters for vlsSMLM filtering.**

Possible optimisation of the filtering

Parameter plots represent a basic way to visualise and threshold the fitted PSFs in a vlsSMLM image. However, other more complex polygons in the parameter plot can be used to even more increase the accuracy of the selection of the in-focus localisations as is commonly used in flow cytometry analysis. In principle other parameters could also be added to an n-dimensional parameter plot, including parameters such as ellipticity, signal-to-noise ratio, etc. (Fig S2). However, in the interests of simplicity, we determine that amplitude and width of the Gaussian fits are the two parameters most able to differentiate selected PSFs from within the vls from out-of-focus ones (Fig 1 and S1).

Filtering width first

The distribution of intensities from single fluorophores in a super-resolution image usually follows a log-normal distribution. The long tail of such a distribution means that the intensity of a fluorophore, whether or not it originates from the focal plane, varies a lot depending on many factors including variables such as its mobility or its dipole orientation. Thus, in a same z plane, the amplitude of a population of fluorophores will follow the same distribution (as the amplitude of a Gaussian is directly proportional to its intensity, at constant width). A bright fluorophore out of the vls has a similar amplitude than a dimmer fluorophore in the vls. Thus it is not optimal to use such a parameter as the only or primary threshold. Similarly, the parameter plots are better spread along the width axis than along the amplitude one (Fig 2). This is why we first apply a width threshold before refining the selection with the amplitude threshold.
