# Supplementary figures and images for: Virtual-'Light-Sheet' Single-Molecule Localisation Microscopy Enables Quantitative Optical Sectioning for Super-Resolution Imaging
Source: PLoS One. 2015 Apr 17;10(4):e0125438. doi: 10.1371/journal.pone.0125438 (PMC4401716; doi:10.1371/journal.pone.0125438)

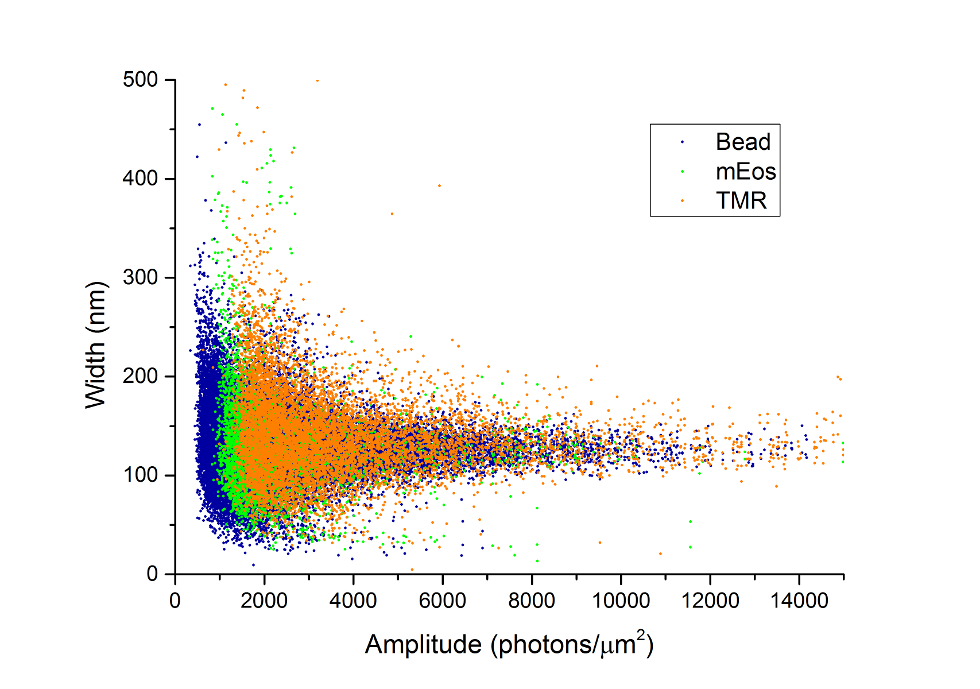

Supplement: S1 Fig — Single fluorophores (TMR* in orange, purified mEos3.1 in green) were fixed on poly-lysine-coated coverslips and imaged under 561 nm illumination. Very low irradiance at 405 nm activation light was used in order to image only a few separated fluorophores per frame. A z-scan was performed so that different fluorophores at different lateral positions were imaged at each axial position of the stage. After fitting each detected 2D-PSF, we rebuilt the width vs. amplitude parameter plot for the localisations detected in the vls and compared it to the one obtained for 505 nm emitting beads (40 nm in diameter) as in Fig 2 (navy blue). The distributions of localisations of the single fluorophores and of the bead in the width vs. amplitude parameter plot are very similar. Imaging beads of diameter ≤ 100 nm under low illumination is consequently an easy way to mimic single fluorophores and calibrate vlsSMLM. (TIF) [file pone.0125438.s002.tif]

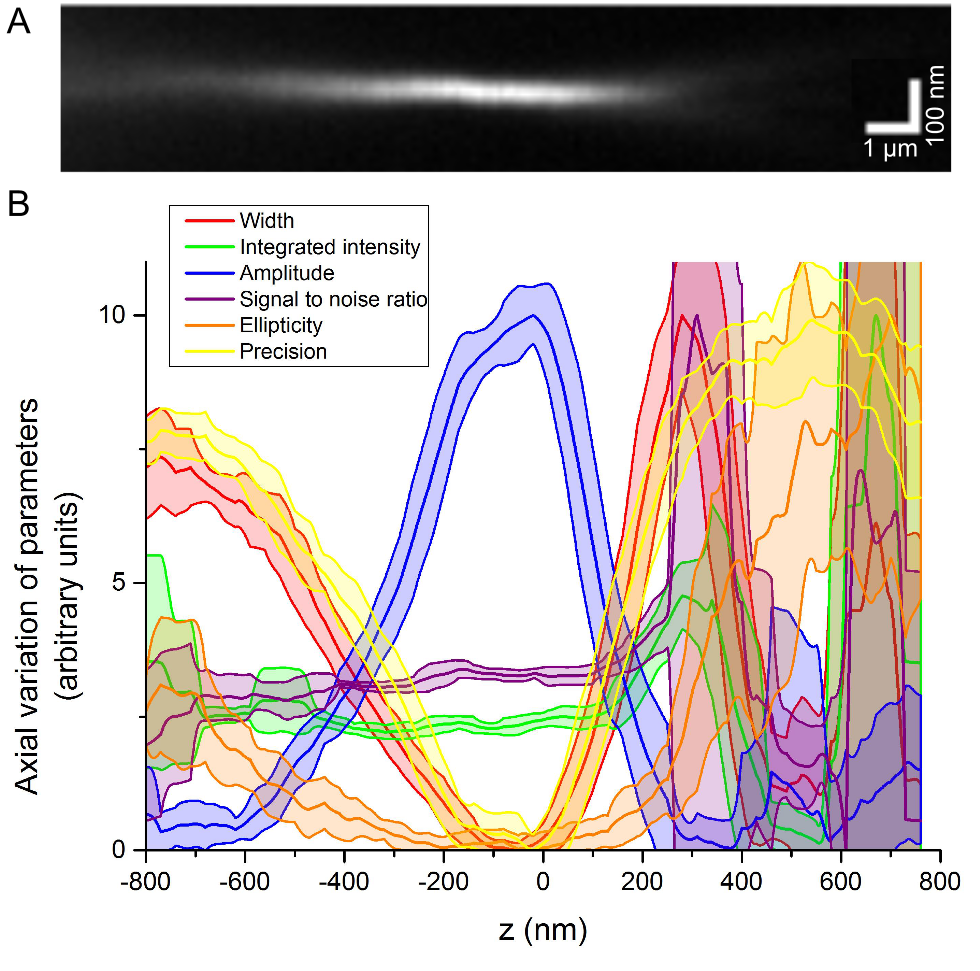

Supplement: S2 Fig — After fitting all detected PSFs from a z-stack of images of beads (Fig 1), the (xz) projection of the sum of 28 bead stacks over 1.6 μm was plotted, (A) showing its axial variation in intensity space. Multiple parameters were considered to define the vls: the amplitude, the integrated intensity, the signal-to-noise ratio, the precision, the width or the ellipticity of the localisations (B). Some parameters, such as the signal-to-noise ratio, the intensity or the ellipticity, do not vary axially and cannot be used to define a precise focal plane. In principle multi-parameter vlsSMLM imaging is possible; however, the amplitude, precision and width were best suited to large variation relative to the focal plane. We chose the width and the amplitude as our vlsSMLM thresholds as their variation at a given plane (shades around each curve: +/- one standard deviation of the mean) were slightly smaller and therefore would lead to a more precise vls. (TIF) [file pone.0125438.s003.tif]

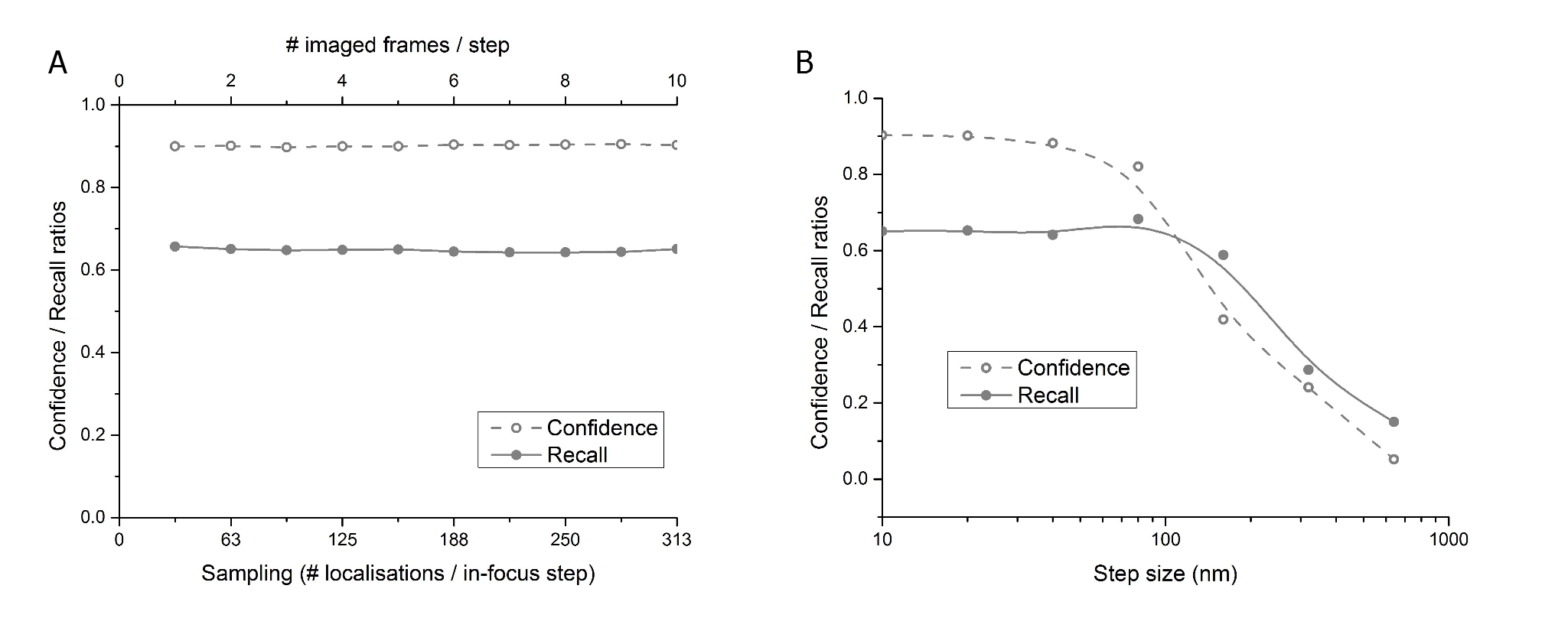

Supplement: S3 Fig — Varying the number of beads fitted per z-step, by changing the number of frames imaged per z-step (A), had no impact on the computed confidence (dotted line) and recall ratios (solid line). As few as 31 localisations per in-focus step are sufficient for a correct sampling of the calibration. However, the size of the step of the z-stack had, as expected, a strong impact on the calibration efficiency (B): the confidence rate (dotted line) dropped importantly as soon as the sampling involved z-steps of more than 40 nm. The recall rate (solid line) was flatter, but also dropped for higher z-step sizes of 80 nm or more. We therefore recommend sampling the calibration beads at least every 40 nm along the optical axis; this can be achieved in multiple ways including piezo, mechanical or even manual positioning. (TIF) [file pone.0125438.s004.tif]

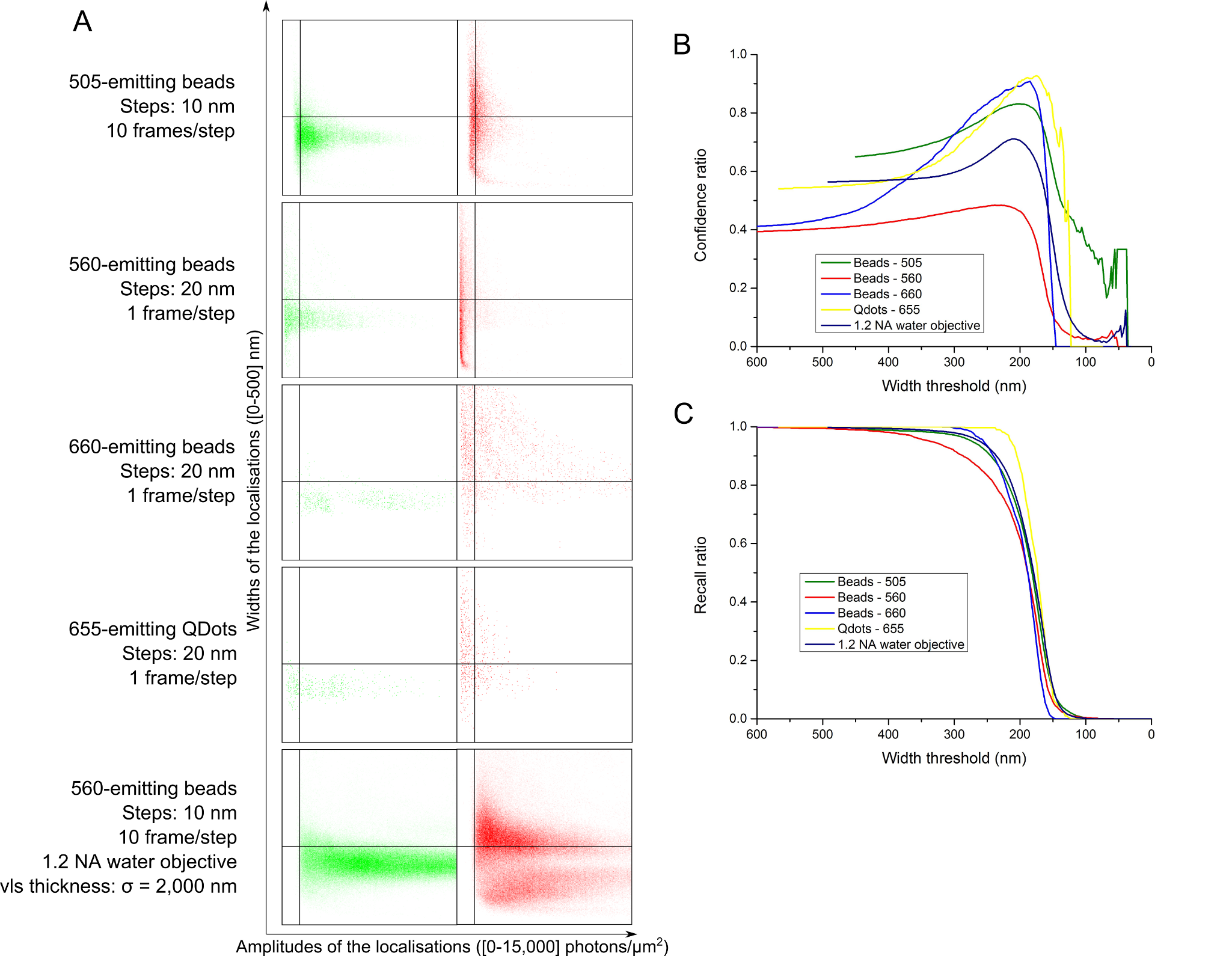

Supplement: S4 Fig — Independent z-scans of different isolated fluorescent particles were imaged on different instruments to test the robustness of the vlsSMLM calibration. Analyses of four z-stacks (see S1 Material and Methods) were compared to the z-stack described in the Results section (see Material and Methods): one frame of 505 nm, 560 nm, or 660 nm emitting beads, or 655 nm emitting quantum dots was imaged every 20 nm on three different instruments; ten frames of 560 nm emitting beads were also imaged every 40 nm with a water immersion 1.2 numerical-aperture objective. The thickness of the vls is anti-correlated with the numerical-aperture objective lens [33]: for the 1.2 numerical-aperture objective, the thickness of the vls was increased to σ = 2,000 nm instead of 283 nm. The parameter plots (A) look relatively similar, although they vary in density; the PSFs from the vls are more dense below the 223 nm width threshold (black horizontal line). Black vertical lines correspond to the 1,502 photons/μm2 amplitude threshold. Similarly, the variations of the confidence (B) and the recall (C) ratios qualitatively follow a similar shape; they however peak (B) and drop (C) at slightly different threshold values depending on both the wavelength of the fluorescent particles and on the specific instrument. This behaviour is experimentally expected as the width of the PSF is wavelength-dependent [20] and because of the imperfect optics used that differently affect the width of the PSF. A calibration with particles whose spectrum is matched with the spectrum of the fluorophores used in the SMLM experiment is therefore recommended, on the very same microscope. Interestingly, the confidence ratio for the 560-emitting beads experiment is globally lower. This is due to some diffusing beads in solution which are out of the vls but are considered in it during the calibration. (TIF) [file pone.0125438.s005.tif]

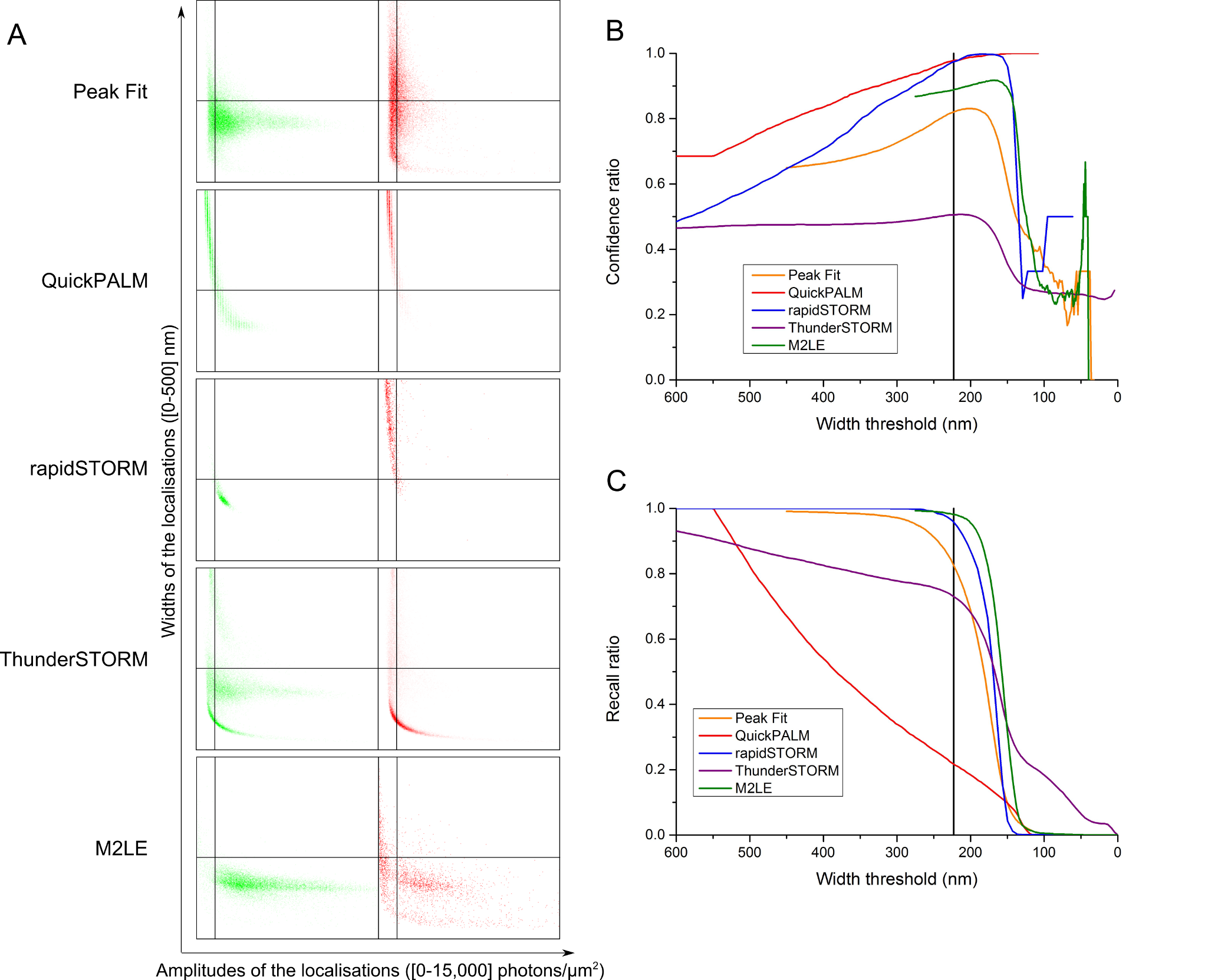

Supplement: S5 Fig — Different, widely used, fitting algorithms were used on the same z-stack of images of beads: Peak Fit [21], QuickPALM [34], rapidSTORM [35], ThunderSTORM [36] and M2LE [37]. Their difference is reflected in the parameter plots that were calculated (A). However, in-focus PSFs (green parameter plots, A) are always found in majority just below the 223 nm width threshold (black horizontal line). The confidence (B) and recall (C) ratio curves for the different fitting algorithms follow a similar shape. They however peak (B) and drop (C) at different values, underlining the importance of analysing the calibration z-scan in the very same way as the SMLM data, using the same fitting routine. The 223 nm width and 1,502 photons/μm2 amplitude thresholds are shown as black horizontal and vertical lines. Outlier behaviour is noticeable for QuickPALM. This could be explained by a low ability of QuickPALM to precisely fit the widths of the PSFs. (TIF) [file pone.0125438.s006.tif]
