# Supplementary material for: Virtual-'Light-Sheet' Single-Molecule Localisation Microscopy Enables Quantitative Optical Sectioning for Super-Resolution Imaging
Source: PLoS One. 2015 Apr 17;10(4):e0125438. doi: 10.1371/journal.pone.0125438 (PMC4401716; doi:10.1371/journal.pone.0125438)
Supplement: S1 Material and Methods — (DOCX) [file pone.0125438.s007.docx]

# Virtual-‘Light-Sheet’ Single-Molecule Localisation Microscopy Enables Quantitative Optical Sectioning for Super-Resolution Imaging.

Matthieu Palayret^1^, Helen Armes^1,2^, Srinjan Basu^3^, Adam T Watson^2^, Alex Herbert^2^, David Lando^3^, Thomas J Etheridge^2^, Ulrike Endesfelder^4^, Mike Heilemann^4^, Ernest Laue^3^, Antony M Carr^2^, David Klenerman^1^, Steven F Lee^1*^

^1^ Department of Chemistry, University of Cambridge, Lensfield Road, Cambridge CB2 1EW, UK

^2^ Genome Damage and Stability Centre, University of Sussex, Falmer, Sussex BN1 9RQ, UK

^3^ Department of Biochemistry, University of Cambridge, 80 Tennis Court Road, Cambridge CB2 1GA, UK

^4^ Institute of Physical and Theoretical Chemistry, Goethe University Frankfurt, Max-von-Laue-Str. 7, 60438 Frankfurt, Germany

* [sl591@cam.ac.uk](mailto:sl591@cam.ac.uk)

#### S1 Material and Methods:

Purification of mEos3.1 (adapted from Fang et al. [1])

The *E.coli* codon optimised mEos3.1 gene was cloned into the N-terminal 6xHis-tagging expression pTWO-E vector (a kind gift from Pearl lab) and the resulting plasmid was transformed into *E. coli* BL21(DE3) cells (Novagen). Three litres of culture were grown to an absorbance of A600 nm 0.6 and cooled to 20^˚^C. Isopropyl b-D-thiogalactopyranoside (IPTG – Fisher Scientific) was then added to a final concentration of 0.5mM to induce protein expression, and the cultures were incubated in a shaker for a further 14–16 h. Collected cell pellets were placed at -20^˚^C overnight (not snap frozen). Pellets were suspended in buffer A (10 mM Tris, 100 mM NaCl, 50 mM Na_2_HPO_4_, pH8.0) with EDTA-free protease inhibitor mix (Roche) (1 tablet/ 3 L of culture) and AEBSF at 10 µg/mL, to a total volume of 35 mL in a 50 mL Falcon tube, and lysed by sonication using a SONICS VIBRA-CELL with 5 s sonication and 10 s rest with a total sonication time of 3 minutes. Lysed cells were centrifuged at 18,500 r.p.m. (32,000g) in Allegra 64R ultracentrifuge (Beckman Coulter) with a F0650 rotor for 1h and the supernatant was applied to a 5 mL Ni-NTA agarose resin (Qiagen) (pre-equilibrated using buffer A) and incubated 1 h at 4^˚^C with gentle agitation. The column was washed with eight column volumes of buffer B (50 mM KH_2_PO_4_, 300 mM NaCl, 20 mM imidazole, 5% glycerol, pH7.9), and the protein was eluted with two column volumes of buffer C (50 mM KH_2_PO_4_, 300 mM NaCl, 250 mM imidazole, 5% glycerol, pH7.9). The protein was further purified on an SD200 size exclusion column pre-equilibrated using buffer D (10 mM Tris, 100 mM NaCl, 5% glycerol, pH8.0 degassed) and protein aliquots snap frozen in liquid N_2_ and stored at -80^˚^C. The protein purification was performed at 4^˚^C. The exposure to light was minimised by covering tubes and columns in foil, using red LED light and growing cultures in the dark.

Sample preparation for Fig S1

Purified mEos3.1 and SNAP-Cell® TMR-Star (New England BioLabs, S9105S) were diluted 10^-5^ in twice-200-nm-filtered PBS and fixed for 5’ on poly-lysine-coated plasma-cleaned coverslips. Coverslips were washed three times with filtered PBS and imaged as described in the Material and Methods, PALM imaging section, using the Semrock, FF01-587/35 emission band-pass filter.

Sample preparation for Fig S4

550 nm emitting TetraSpeck beads (Invitrogen) were fixed onto a coverslip and imaged every 20 nm using a z piezo-stage on a replica of the instrument described in the Material and Methods. The fluorescence signal was spectrally separated from the illumination light by a 561 long-pass (Semrock, BLP01-561R) and a band-pass filters (Semrock, FF01-593/40). The exposure of each frame was set to 100 ms.

680 nm emitting TetraSpeck beads (Invitrogen) or 655 nm emitting quantum dots were fixed on a coverslip. Chamber slides were mounted on a Nikon Ti Eclipse inverted microscope equipped with 100x oil immersion objective lens (Apo TIRF 100X 1.49 Oil, Nikon). The 647 nm beam of a multiline argon krypton mixed gas ion laser (Innova70C, Coherent) was projected into the microscope on a quad-band dichroic mirror (F73-888, AHF). Fluorescence light was collected by the objective, spectrally separated from the excitation light by a band-pass filter (F47-700, AHF) and projected on the 512 x 512 pixel chip of an EMCCD (iXon DU897, Andor). Videos of the traces of the different fluorescent particles were recorded at different axial position in 20 nm steps using a z piezo stage.

## References:

1. Fang C, Frontiera RR, Tran R, Mathies RA. Mapping GFP structure evolution during proton transfer with femtosecond Raman spectroscopy. Nature. Macmillan Publishers Limited. All rights reserved; 2009;462: 200–4. doi:10.1038/nature08527
