# Supplementary material for: Virtual-'Light-Sheet' Single-Molecule Localisation Microscopy Enables Quantitative Optical Sectioning for Super-Resolution Imaging
Source: PLoS One. 2015 Apr 17;10(4):e0125438. doi: 10.1371/journal.pone.0125438 (PMC4401716; doi:10.1371/journal.pone.0125438)
Supplement: S1 Table — (DOCX) [file pone.0125438.s010.docx]

# Virtual-‘Light-Sheet’ Single-Molecule Localisation Microscopy Enables Quantitative Optical Sectioning for Super-Resolution Imaging.

Matthieu Palayret^1^, Helen Armes^1,2^, Srinjan Basu^3^, Adam T Watson^2^, Alex Herbert^2^, David Lando^3^, Thomas J Etheridge^2^, Ulrike Endesfelder^4^, Mike Heilemann^4^, Ernest Laue^3^, Antony M Carr^2^, David Klenerman^1^, Steven F Lee^1*^

^1^ Department of Chemistry, University of Cambridge, Lensfield Road, Cambridge CB2 1EW, UK

^2^ Genome Damage and Stability Centre, University of Sussex, Falmer, Sussex BN1 9RQ, UK

^3^ Department of Biochemistry, University of Cambridge, 80 Tennis Court Road, Cambridge CB2 1GA, UK

^4^ Institute of Physical and Theoretical Chemistry, Goethe University Frankfurt, Max-von-Laue-Str. 7, 60438 Frankfurt, Germany

* [sl591@cam.ac.uk](mailto:sl591@cam.ac.uk)

S1 Table: **DNA primers used for Cenp-A-mEos3.2 cloning and sequencing.**

| NcoI_mEos3_F | TGAACACGTGGCCAccatggGTAGTGCGATTAAGCCAGACATGAAGATCA |
| --- | --- |
| XhoI_mEos3_R | AGGctcgagTCGTCTGGCATTGTCAGGCAATC |
| XhoI_NheI_Cenp-A_F | AGGctcgaggctagcATGGGCCCGCGTCGCAAA |
| Xba1_Cenp-A_R | AGGtctagaTTAGGGGAGTCCGCCCTCG |
| pEF_F | TCTCAAGCCTCAGACAGTGGTTCAAAGT |
| BGH_R | TAG AAG GCA CAG TCG AGG |
